# Airborne particulate matter and mitochondrial damage: a cross-sectional study

**DOI:** 10.1186/1476-069X-9-48

**Published:** 2010-08-09

**Authors:** Lifang Hou, Zhong-Zheng Zhu, Xiao Zhang, Francesco Nordio, Matteo Bonzini, Joel Schwartz, Mirjam Hoxha, Laura Dioni, Barbara Marinelli, Valeria Pegoraro, Pietro Apostoli, Pier Alberto Bertazzi, Andrea Baccarelli

**Affiliations:** 1 Department of Preventive Medicine, Feinberg School of Medicine, Northwestern University, Chicago, Illinois, USA; 2 Robert H. Lurie Comprehensive Cancer Center Feinberg School of Medicine, Northwestern University, Chicago, Illinois, USA; 3 Center of Molecular and Genetic Epidemiology, Department of Environmental and Occupational Health, University of Milan and IRCCS Maggiore Policlinico Hospital, Mangiagalli and Regina Elena Foundation, Milan, Italy; 4 Department of Oncology, No.3 People's Hospital, School of Medicine, Shanghai Jiaotong University, Shanghai, China; 5 Department of Clinical Medicine, Nephrology and Health Sciences, University of Parma Medical School, Parma, Italy; 6 Department of Clinical and Biological Sciences, University of Insubria, Varese, Italy; 7 Exposure, Epidemiology and Risk Program, Department of Environmental Health, Harvard School of Public Health, Boston, Massachusetts, USA; 8 Department of Experimental and Applied Medicine, Occupational Medicine and Industrial Hygiene, University of Brescia, Brescia, Italy

## Abstract

**Background:**

Oxidative stress generation is a primary mechanism mediating the effects of Particulate Matter (PM) on human health. Although mitochondria are both the major intracellular source and target of oxidative stress, the effect of PM on mitochondria has never been evaluated in exposed individuals.

**Methods:**

In 63 male healthy steel workers from Brescia, Italy, studied between April and May 2006, we evaluated whether exposure to PM was associated with increased mitochondrial DNA copy number (MtDNAcn), an established marker of mitochondria damage and malfunctioning. Relative MtDNAcn (RMtDNAcn) was determined by real-time PCR in blood DNA obtained on the 1^st ^(time 1) and 4^th ^day (time 2) of the same work week. Individual exposures to PM_10_, PM_1_, coarse particles (PM_10_-PM_1_) and airborne metal components of PM_10 _(chromium, lead, arsenic, nickel, manganese) were estimated based on measurements in the 11 work areas and time spent by the study subjects in each area.

**Results:**

RMtDNAcn was higher on the 4^th ^day (mean = 1.31; 95%CI = 1.22 to 1.40) than on the 1^st ^day of the work week (mean = 1.09; 95%CI = 1.00 to 1.17). PM exposure was positively associated with RMtDNAcn on either the 4^th ^(PM_10_: β = 0.06, 95%CI = -0.06 to 0.17; PM_1_: β = 0.08, 95%CI = -0.08 to 0.23; coarse: β = 0.06, 95%CI = -0.06 to 0.17) or the 1^st ^day (PM_10_: β = 0.18, 95%CI = 0.09 to 0.26; PM_1_: β = 0.23, 95%CI = 0.11 to 0.35; coarse: β = 0.17, 95%CI = 0.09 to 0.26). Metal concentrations were not associated with RMtDNAcn.

**Conclusions:**

PM exposure is associated with damaged mitochondria, as reflected in increased MtDNAcn. Damaged mitochondria may intensify oxidative-stress production and effects.

## Background

Ambient and occupational exposure to Particulate Matter (PM) has been linked with increased hospitalization and mortality from cardiovascular disease, respiratory disease, and lung cancer [1-8]. *In-vitro *and *in-vivo *studies have identified generation of reactive oxygen species (ROS) and increased oxidative stress as a primary biological process that may contribute to produce such a variety of diverse health effects [9-14]. Epidemiologic [1,15] and *in-vivo *studies [16-18] suggest that both PM mass and PM metal components contribute to ROS generation. Mitochondria are the major intracellular source and primary target of ROS, which are generated under normal conditions as by-products of aerobic metabolism in animal and human cells. Each human and animal cell contains between several hundred and over a thousand mitochondria, each carrying 2-10 copies of mitochondrial DNA (MtDNA). MtDNA copy number (MtDNAcn) is correlated with the size and number of mitochondria, which have been shown to change under different energy demand, as well as different physiological or environmental conditions [19].

Compared with nuclear DNA, MtDNA lacks protective histones and has diminished DNA repair capacity, and is therefore particularly susceptible to ROS-induced damage [20]. Cells challenged with ROS have been shown to synthesize more copies of their MtDNA and increase their mitochondrial abundance to compensate for damage and meet the increased respiratory demand required for ROS clearance [19]. At the same time, ROS are also generated from the increased mitochondria in these cells and thus cause additional oxidative damage to mitochondria and other intracellular constituents including DNA, RNA, proteins, and lipids [21]. Inter-individual variations of cellular MtDNAcn are observed in the general population [22]. In a study by Liu et al., MtDNAcn in circulating blood leukocytes was positively correlated with blood markers of oxidative stress [23]. Mitochondrial damage and dysfunction, as reflected in increased MtDNAcn, may thus represent a biological effect along the path linking PM inhalation to its health effects. However, whether MtDNAcn is increased in individuals exposed to airborne PM has never been evaluated.

Foundry work has been associated with various adverse health outcomes, including cardiovascular disease, respiratory disease, and lung cancer, that may depend on ROS-induced damage and genotoxicity [24-26]. Even in modern foundry facilities that adopt state-of-art measures for exposure reduction, workers are still exposed to substantially higher levels of airborne PM compared to those found outdoors [27]. In the present study on foundry workers exposed to a wide range of PM levels, we investigated the association of blood MtDNAcn with exposures to airborne PM and its metal components.

## Methods

### Subjects

We recruited 63 male healthy workers (mean age = 44 years, ranging between 27 and 55 years), free of cancer and cardiovascular and pulmonary disease, in an electric steel plant in Brescia, Northern Italy between April and May 2006. Twenty-five subjects (40%) were current smokers, who reported a mean duration of smoking of 13.0 years (SD = 12). Most subjects had middle school (59%) or higher (22%) education and lived in suburban areas (67%). The average body mass index (BMI) was 26.5 kg/m^2 ^(SD = 2.7). The distribution of self-reported traffic intensity near home was 8% for high, 62% for medium, and 30% for low. All participants had been working in the current job position for at least one year. A self-administered questionnaire was used to collect detailed information on lifestyle, drug use, recent medical conditions, and residential history. Records from the factory administrative and clinical files were used to abstract information on occupational and past medical history.

In order to discriminate short- and long-term effects of PM, we obtained blood samples at two different times: i) the time 1 sample was collected in the morning of the 1^st ^day of a work week (following two days off work) before the beginning of any work activity; ii) the time 2 sample was collected at the same hour on the 4^th ^work day of the same week. Individual written informed consent and approval from the local Institutional Review Board were obtained before the study.

### Exposure Measurement

Measures of the airborne levels of PM mass and PM metal components obtained in each of the 11 work areas of the plant were used to estimate individual exposures. During the three work days between times 1 and 2, each of the study subjects recorded the time that he spent in each of the work areas. For each of the exposures, we calculated the personal time-weighted average exposure level by multiplying the time spent in each area by the level of PM mass or PM metal components in the area, which was then divided by the total time spent at work. The exposure levels of each pollutant in each of the work areas have shown very little variability over time, as measures repeated over one year in a subset of subjects showed very high correlation (*r*^2 ^> 0.90). Because all the study subjects reported in the questionnaire to have performed their standard work routine during the three days of the study, the time-weighted personal levels of PM mass and PM metal components, in addition to the exposure during the week of the study, were also a measure of the usual exposure of the study subjects [27].

Measures of PM mass included concentrations of airborne PM with aerodynamic diameters < 10 μm (PM_10_) and < 1 μm (PM_1_) measured using a GRIMM 1100 light-scattering dust analyzer (Grimm Technologies, Inc. Douglasville, GA, USA). Concentrations of coarse particles were calculated from these measures as the difference between PM_10 _and PM_1_. Measures of PM metal components were performed on the PM_10 _fraction of PM mass through multi-elemental analysis by means of inductively coupled-plasma mass spectrometer (ICP-MS, ELAN DRC II, Perkin Elmer, Waltham, USA). We measured arsenic, lead, manganese, and nickel concentrations using the Total Quant method, whereas chromium concentrations were measured using the Dynamic Reaction Cell (DRC) method with ammonia. External calibration was performed using calibration standard 3, stock multi-element (10 μg/ml; Perkin Elmer, Waltham, USA). The coefficient of variations for the metal concentrations obtained in repeated measures varied between 4% and 8%.

### Measurement of MtDNAcn

We used EDTA tubes to collect 7 ml whole blood that was promptly centrifuged on site at 2500 rpm for 15 minutes. The buffy coat (400 μl) was transferred in a cryovial, immediately frozen in vapour phase of liquid nitrogen, and shipped in nitrogen dry shippers to the laboratory. DNA was extracted using the Wizard Genomic DNA purification kit (Promega, Madison, WI) following the manufacturer's instructions. The samples collected in the 1^st ^and 4^th ^day were processed using the same exact protocols.

Relative MtDNAcn (RMtDNAcn) was measured in buffy coat DNA by a quantitative real time polymerase chain reaction (PCR) assay that measure relative mitochondrial copy number by determining the ratio of mitochondrial (Mt) copy number to single copy gene (S) copy number in experimental samples relative to a reference [28]. This method is based on quantification of Mt and S quantities expressed as Cts derived from a standard curve obtained from serial dilutions of a reference DNA. The reference single copy gene used in this study was human [beta] globin (hbg). The Mt PCR mix was: iQ SYBR Green Supermix (Bio-Rad) 1×, MtF3212 500 nM, MtR3319 500 nM, EDTA 1×. The S (hbg) PCR mix was: iQ SYBR Green Supermix (Bio-Rad) 1×, hbgF 500 nM, hbgR 500 nM, EDTA 1×. 9 ng DNA was loaded in a 20 μl PCR reaction. We used pooled DNA from 20 participants randomly selected from this same study (500 ng for each sample) to create in every Mt and S PCR run a fresh standard curve, which ranged from 20 ng/μl to 0.25 ng/μl. The primers for RT Q-PCR analysis of MtDNA were: MtF3212 5'CAC CCA AGA ACA GGG TTT GT3', and MtR3319 5'TGG CCA TGG GTA TGT TGT TA3'. The primers for RT Q-PCR analysis of hbg were: hbgF 5'GCT TCT GAC ACA ACT GTG TTC ACT AGC3', and hbgR 5'CAC CAA CTT CAT CCA CGT TCA CC3'. All PCRs were performed on a DNA Engine thermal cycler Chromo4 (Bio-Rad, Hercules, California, USA). The thermal cycling conditions for MtDNA PCR were: initial 2 minutes at 50°C, and 3 minutes at 95°C to activate the hot-start iTaq DNA polymerase, followed by 40 cycles comprised of 15 s denaturation at 95°C and 49 s anneal/extend at 60°C. The thermal cycling conditions for the hbg PCR were 3 minutes at 95°C to activate the hot-start iTaq DNA polymerase, followed by 40 cycles comprised of 15 s denaturation at 95°C and 49 s anneal/extend at 58°C. Each run was completed by melting curve analysis to confirm the amplification specificity and absence of primer dimers.

All samples were run in triplicates. The average of the three Mt measurements was divided by the average of the three S measurements to calculate the Mt/S ratio for each sample. For quality control purposes, 10 blind duplicate samples were interdispersed among the test samples. The coefficient of variation for the Mt/S ratio in duplicate samples was 3.2%.

### Statistical Analysis

The paired student's t-test was used to assess differences in RMtDNAcn measured on the 1^st ^day of a work week (time 1) and RMtDNAcn measured on the 4^th ^days in the same week (time 2). We used linear regression models to evaluate the association of personal time-weighted average levels of the individual exposures with RMtDNAcn. We fit different sets of models to evaluate the association of individual exposures with: A) RMtDNAcn measured at time 1; B) RMtDNAcn measured at time 2; and C) the difference in RMtDNAcn between time 2 and time 1. The personal time-weighted average levels of exposures were estimated based on measurements of the pollutant concentrations in the plant work areas that were done between time 1 and time 2. However, as specified in the exposure measurement section above, the time-weighted personal levels were also a measurement of the usual exposure of the study subjects, as demonstrated by the high within-subject correlation of the exposure levels measured in different weeks over one year. The model set A was therefore run to evaluate potential permanent effects associated with the usual exposure of the study subjects that could be more easily detected on the time 1 samples (which were collected after two days off work).

All models in each of the sets were adjusted for age, BMI, smoking status, years of smoking and percent neutrophils in blood count. To allow for comparison of the size of effects from different exposures, we report all model results as beta coefficients estimating the change in RMtDNAcn due to an increment in exposure equal to the difference between the 90^th ^and 10^th ^percentile. As a sensitivity analysis, we repeated all statistical analyses using the logarithm of RMtDNAcn. Results using log (RMtDNAcn) did not show meaningful departures from those obtained using RMtDNAcn on the original scale. Results based on RMtDNAcn on the original scale are reported throughout the paper. All analyses were performed in Stata 10.0 (Stata Corp., College Station, TX).

## Results

Table 1 shows the distributions of the personal time-weighted average exposure to PM_10_, PM_1_, coarse particles, as well as of the metals contained in the PM_10 _fraction estimated during the three work days between the times 1 and 2 RMtDNAcn measurements. Average levels of PM exposure varied between 73.40-1220.17 μg/m^3 ^with a mean of 233.42 μg/m^3 ^(SD = 215.00) for PM_10_, between 1.71-30.49 μg/m^3 ^with a mean of 8.48 μg/m^3 ^(SD = 6.18) for PM_1_, and between 71.51-1189.68 μg/m^3 ^with a mean of 224.94 μg/m^3 ^(SD = 208.99) for coarse particles. Personal time-weighted average exposure levels for metal components ranged between 0.02-0.20 μg/m^3 ^with a mean of 0.09 μg/m^3 ^(SD = 0.03) for chromium; between 0.52-18.00 μg/m^3 ^with a mean of 6.98 μg/m^3 ^(SD = 6.63) for lead; between 0.005-0.500 μg/m^3 ^with a mean of 0.18 μg/m^3 ^(SD = 0.21) for arsenic; between 0.10-0.90 μg/m^3 ^with a mean of 0.43 μg/m^3 ^(SD = 0.26) for nickel; and between 0.30-684.00 μg/m^3 ^with a mean of 38.78 μg/m^3 ^(SD = 98.86) for manganese.

**Table 1:** Time-weighted average levels of exposure to airborne particulate matter and metals among foundry workers in an electric steel plant.

**Pollutant (μg/m**^**3**^)	Mean	SD	Minimum		Percentile		Maximum
					
				**25**^**th**^	**50**^**th**^	**75**^**th**^	
PM_10_	233.42	215.00	73.40	152	179.44	223.00	1220.17

PM_1_	8.48	6.18	1.71	3.51	9.01	11.35	30.49

Coarse	224.94	208.99	71.51	148.60	170.13	211.03	1189.68

Chromium*	0.09	0.03	0.02	0.08	0.09	0.10	0.20

Lead*	6.98	6.63	0.52	1.40	4.22	16.11	18.00

Arsenic*	0.18	0.21	0.005	0.016	0.072	0.500	0.500

Nickel*	0.43	0.26	0.10	0.24	0.34	0.62	0.90

Manganese*	38.78	98.86	0.30	1.33	9.12	30.00	684.00

* Metals components were measured in PM_10_.

**Figure 1:**
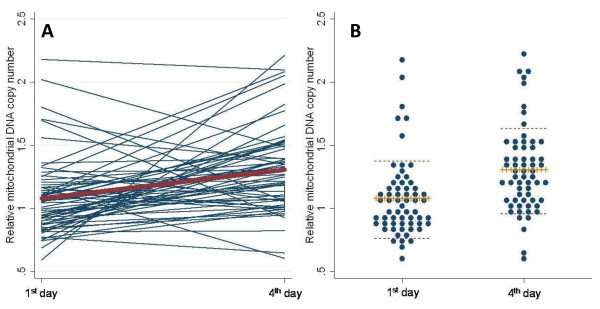
**Relative mitochondrial DNA copy number measured on blood samples collected on the 1^st ^day and 4^th ^day of the work week**. The graphs show the changes of individuals' MtDNA levels (blue lines) and the mean change (bolded red line) between the 1^st ^and 4^th ^day (panel A); and the individual values of MtDNA (blue dot) and the corresponding means (central crossed bar) and standard deviations (shoulder dotted bar) for day 1^st ^and 4^th ^(panel B).

RMtDNAcn was not associated with the characteristics of the study subjects, including age, BMI, smoking duration, duration of employment, education, and area of residence (Table 2). Overall, RMtDNAcn was higher on the 4^th ^work day (time 2: mean = 1.31; 95% CI = 1.22 to 1.40) compared with the RMtDNAcn measured on the 1^st ^day of the same week (time 1: mean = 1.09; 95% CI = 1.00 to 1.17) (Fig. 1B). The correlation between RMtDNAcn measures on day 1^st ^and day 4^th ^was small (Intra-class correlation coefficient = 0.13).

**Table 2:** Relative mitochondrial DNA copy number (RMtDNAcn) by subjects' characteristics.

Variable	n	Mean RMtDNAcn*	95% CI*
Age, years			

< 39	21	1.14	0.96 to 1.33

39 - 47	21	1.29	1.10 to 1.47

> 47	21	1.14	0.95 to 1.32

Body mass index, kg/m^2^			

≤25	21	1.25	1.07 to 1.44

25 - 27.5	21	1.20	1.10 to 1.38

> 27.5	21	1.12	0.93 to 1.30

Smoking duration, years			

0	23	1.17	0.99 to 1.35

0 - 19	19	1.17	0.98 to 1.36

> 19	21	1.23	1.04 to 1.42

Duration of employment, years			

< 9	20	1.27	1.08 to 1.46

9 - 21	20	1.17	0.99 to 1.36

> 21	19	1.15	0.96 to 1.34

Education			

Primary school	12	1.28	1.07 to 1.48

Middle school	37	1.19	1.01 to 1.36

High school	14	1.12	0.92 to 1.32

Area of residence			

Rural	12	1.24	1.05 to 1.42

Suburbs	41	1.14	0.99 to 1.30

City centre	8	1.21	1.00 to 1.41

Self-reported traffic intensity near home			

Low	18	1.12	0.94 to 1.29

Medium	38	1.19	1.03 to 1.35

High	5	1.23	1.00 to 1.46

* Based on measurements of DNA from blood samples taken on the first day of a working week (time 1) and after three consecutive days of work (time 2). As each study subject had two measurements of relative mitochondrial DNA copy number (RMtDNAcn), mixed regression models were used to account for within-subject correlation and estimate means and 95% confidence intervals (CI).

In both unadjusted and covariate-adjusted regression analysis (Table 3), exposures to PM_10_, PM_1_, and coarse particles were associated with RMtDNAcn measured at time 1 (β = 0.18, SE = 0.04, 95% CI = 0.09 to 0.26 for PM_10_; β = 0.23; SE = 0.06, 95% CI = 0.11 to 0.35 for PM_1_; and β = 0.17, SE = 0.04, 95% CI = 0.09 to 0.26 for coarse particle exposures in the multivariable models adjusting for age, BMI, smoking status, and years of smoking). We also observed positive associations of RMtDNAcn measured at time 2 with PM10, PM_1_, and coarse particles (PM_10_: β = 0.06, SE = 0.06, 95% CI = -0.06 to 0.17; PM_1_: β = 0.08, SE = 0.08, 95% CI = -0.08 to 0.23; coarse: β = 0.06, SE = 0.06, 95% CI = -0.06 to 0.17), although the effect size was lower than at time 1. PM and several metal components were negatively correlated with the difference in RMtDNAcn between time 1 and time 2 (Table 3). Analyses on the associations of PM exposure with RMtDNAcn stratified by age, BMI, smoking duration, duration of employment, education and area of residence did not show any modification of PM effect due to those variables (data not shown).

**Table 3:** Associations of the levels of exposure to airborne particulate matter and metals with blood relative mitochondrial DNA copy number measured on the 1^st ^day and the 4^th ^day of a work week.

Exposure	First day of a work week			Fourth day of a work week			Difference		
			
	Beta*	95% CI	p-value	Beta*	95% CI	p-value	Beta*	95% CI	p-value
Unadjusted regression									

*PM*_10_	0.17	0.08 to 0.26	< 0.001	0.06	-0.05 to 0.17	0.27	-0.10	-0.24 to 0.03	0.002

*PM*_1_	0.20	0.07 to 0.32	0.002	0.09	-0.06 to 0.24	0.25	-0.11	-0.30 to 0.08	0.005

*Coarse particles*	0.16	0.07 to 0.26	< 0.001	0.06	-0.05 to 0.17	0.27	-0.10	-0.24 to 0.03	0.003

*Chromium*	0.06	-0.12 to 0.23	0.52	-0.10	-0.31 to 0.10	0.32	-0.16	-0.43 to 0.10	0.73

*Lead*	0.15	-0.04 to 0.34	0.13	-0.15	-0.39 to 0.10	0.25	-0.34	-0.66 to -0.03	0.97

*Arsenic*	-0.002	-0.18 to 0.18	0.98	-0.05	-0.28 to 0.17	0.65	-0.08	-0.37 to 0.22	0.63

*Nickel*	-0.05	-0.25 to 0.15	0.60	-0.05	-0.31 to 0.21	0.72	-0.02	-0.36 to 0.33	0.45

*Manganese*	-0.04	-0.12 to 0.03	0.24	-0.002	-0.09 to 0.09	0.97	0.04	-0.08 to 0.16	0.45

Adjusted regression^+^									

*PM*_10_	0.17	0.08 to 0.27	0.001	0.05	-0.07 to 0.17	0.42	-0.12	-0.27 to 0.03	0.10

*PM*_1_	0.22	0.09 to 0.36	0.001	0.07	-0.09 to 0.24	0.41	-0.15	-0.35 to 0.05	0.14

*Coarse particles*	0.17	0.08 to 0.26	0.001	0.05	-0.07 to 0.17	0.42	-0.12	-0.26 to 0.03	0.10

*Chromium*	0.11	-0.09 to 0.31	0.26	-0.15	-0.39 to 0.09	0.22	-0.27	-0.59 to 0.05	0.01

*Lead*	0.14	-0.06 to 0.35	0.17	-0.08	-0.36 to 0.19	0.55	-0.26	-0.61 to 0.08	0.13

*Arsenic*	-0.04	-0.24 to 0.16	0.71	0.03	-0.23 to 0.29	0.79	0.05	-0.28 to 0.39	0.76

*Nickel*	-0.05	-0.27 to 0.17	0.65	0.03	-0.26 to 0.32	0.82	0.06	-0.30 to 0.43	0.74

*Manganese*	-0.06	-0.16 to 0.04	0.21	-0.001	-0.11 to 0.11	0.98	0.12	-0.07 to 0.32	0.21

* Beta for an increment equal to the difference between the 90^th ^and 10^th ^percentile of pollutant.

^+ ^Adjusted for age, body mass index, smoking status, year of smoking and percent neutrophils in blood count. Multivariable analyses based on the RmtDNAcn measured on the forth day of a work week were also adjusted for the level of RMtDNAcn on the first day, in addition to age, body mass index, smoking status, year of smoking and percent neutrophils in blood count. Results for the forth day of a work week from multivariable models including only age, body mass index, smoking status, year of smoking and percent neutrophils in blood count as independent variables did not show major changes from those shown here.

## Discussion

Extensive evidence indicates that generation of oxidative stress contributes to the health effects associated with ambient and occupational PM exposure [10-12]. In our previous study, we found an alteration in blood DNA methylation in a population of foundry works with long-term exposure to PM. Further studies are required to determine the effects of PM on molecular responses to PM exposure, and RMtDNAcn is one of the potential mechanisms affected by the exposure. MtDNAcn is more prone to oxidative damage than the nuclear genome, and increases in MtDNAcn in response to oxidative damage result from a feedback response that compensates for defective mitochondria bearing impaired respiratory chain or mutated MtDNA [28]. In this context, the positive association in the present study of PM exposures with MtDNAcn could be the result of a cellular response to the increased oxidative stress caused by exposure to PM. This response has been suggested to have a dual role in cells challenged by oxidative stress. On one hand, it stimulates mitochondrial proliferation to supply energy to meet the need for cell survival, including repair of damage and synthesis of new proteins. On the other hand, the increasing abundance of dysfunctional mitochondria causes excess ROS production and further oxidative damage that may initiate cell senescence or death [29].

In our study, RMtDNAcn was higher on the 4^th ^day than the one on the 1^st ^day of the same work week, but this increase was inversely associated with the average personal exposure levels between the two RMtDNAcn measurements. It is worth noting that the small correlation of RMtDNAcn measures between the 1^st ^and 4^th ^day samples (Intra-class correlation coefficient = 0.13) may have resulted from the large difference in RMtDNAcn found between the two samples in our data. Future studies in non-exposed subjects are needed to estimate the stability of RMtDNAcn levels over longer time periods.

We also found that the association between personal time-weighted average levels of PM exposure and RMtDNAcn was stronger on the 1^st ^day relative to the 4^th ^day of the work week. Taken together, these findings suggest that the correlations between PM exposure and RMtDNAcn were the result of protracted, long-term exposure to PM. In fact, the RMtDNAcn on the 1^st ^day of work was measured after two days off work as a baseline measure in which potential short-lived influences of the exposures occurred during the previous week were washed out. It is important to consider that in our study the personal time-weighted average of PM levels reflected not only the exposure during the work week of the study, but was also highly correlated with the usual exposure of the study subjects [27]. Therefore, the association of PM exposure levels with RMtDNAcn in the 1^st ^day samples would reflect an association with usual exposure, rather than with that measured during the week of the study. The outcome of the increase of RMtDNAcn is dependent on oxidative stress level, cell antioxidant capacity and quality of mitochondria and MtDNA [29]. Thus, mild oxidative stress may stimulate RMtDNAcn synthesis, while long-term high exposure may result in decreased or no synthesis, owing to severe oxidative damage of cells. Therefore, we hypothesized that the permanent increase in RMtDNAcn due to long-term high levels of exposure would make the highly exposed subjects less sensitive to the acute exposure, while subjects with average lower exposures, who had lower RMtDNAcn on the 1^st ^day of the work week, would exhibit more increase in their RMtDNAcn in response to the exposure during the week of the study. This hypothesis was supported by the inverse correlations we observed between the exposures we measured and the difference in RMtDNAcn between the 4^th ^and the 1^st ^days, which indicate that the increase in RMtDNAcn occurred after three days of exposure was stronger or even limited to the subjects with lower exposure.

Alternatively, it is possible that other unmeasured exposures might have been responsible for the increased RMtDNAcn during the week of the study. In addition to PM, workers in foundries may have additional exposures, including heat, polycyclic aromatic hydrocarbons [30], carbon monoxide [31], and non-ionizing radiations [32], which could also contribute to generate oxidative stress.

Some of previous investigations have suggested that MtDNA has a long-half life and low turnover rates. For instance, Collins et al showed a half-life of approximately 350 days for MtDNA in rat heart tissues by using a stable isotope-mass spectrometric technique [33]. However, other studies have shown more rapid half-lives, varying between 7 and 31 days [34]. It has been postulated that the MtDNA turnover rates are variable depending on the tissue investigated as well as due to the effects of environmental factors [34,35]. Taken together, these data are consistent with our hypothesis on the presence of both rapid and slow changes in RMtDNAcn.

To the best of our knowledge, this is the first study reporting the association between MtDNAcn and PM exposure. Our findings add new information to previous *in-vitro *experiments, which have demonstrated that particles cause ROS-mediated structural alterations in the mitochondrion, including extensive disruption of mitochondrial cristae and vacuolar cellular appearance [12,36-38]. Because of the limited number of study subjects, it is possible that the associations observed were due to confounding or chance. We tried to control confounding by adjusting for different covariates in difference scales but adjustment for none of them changed the association substantially. However, the confounding effect of other time dependent factors such as diet, alcohol use or sunlight, which were unavailable in the present study, cannot be excluded. Our study was based on occupational PM-exposed subjects working in several work areas with relatively controlled environment of the same factory and did not include a different population of subjects without a specific condition of exposure to PM. Limiting our investigation to individuals who have all been working in the same work facility avoided potential concerns related to the selection of external referents who might have differed from the exposed population in terms of socioeconomic factors and other characteristics determining hiring into the plant [39]. However, the differences in the individual levels of PM exposure in our study group were large, providing sufficient contrast for identifying exposure-related changes in MtDNAcn. For example, the lowest level of PM_10 _observed in our study population (73.4 μg/m^3^) was only marginally higher than ambient PM_10 _levels measured in the geographic area in which the plant is located [average annual ambient PM_10 _levels between 41 and 57 μg/m^3 ^were recorded in the year of the study by different ambient monitoring stations in the Brescia area] [40], and the highest levels was 1,220 μg/m^3^. In the present study we measured RMtDNAcn on buffy coat from peripheral blood, which contains both blood leukocytes and platelets. Because platelets have been shown to account for a large proportion of mitochondrial DNA content in blood DNA [41], it is possible that_PM-related changes in RmtDNA copy number are associated with platelet activation induced by the exposures [42,43].

MtDNA has been shown to change in response to other environmental exposures. For example, Shen found an association between MtDNAcn increase and ROS caused by benzene exposure [44]. The possible mechanisms underlining this association include increased membrane permeability or induced apoptosis caused by ROS. Many signalling pathways have been shown to be activated by excess production of ROS, such as AP1and NF-KB [45], p38 MAPK [46], and PI3K-AKT [47]. Although significant advances have been made in understanding the role of MtDNAcn and ROS in health, future research is warranted to better elucidate the pathways that might link exposure-generated ROS with health-related outcomes.

In summary, we observed a potential long-term effect of PM_10_, PM_1_, and coarse particles on MtDNAcn in a group of foundry workers who, in general, had higher levels of exposure to PM compared to the ambient PM levels the general populations are exposed to. These findings suggest that increased oxidative damage of mitochondrial DNA, as reflected in peripheral blood MtDNAcn, may play a critical role in the etiology of PM-related diseases. Further studies are required to validate the present findings as well as to better elucidate the time-relationships between PM-exposure and MtDNAcn.

## Conclusions

PM exposure is associated with damaged mitochondria, as reflected in increased MtDNAcn. Damaged mitochondria may intensify oxidative-stress production and effects.

## List of Abbreviations

BMI: body mass index; MTDNA: mitochondrial DNA; MTDNACN: mitochondrial DNA copy number; RMTDNACN: relative mitochondrial DNA copy number; PCR: polymerase chain reaction; PM: particulate matter; ROS: reactive oxygen species.

## Competing interests

The authors declare that they have no competing interests.

## Authors' contributions

LH and AB generated the study concept, directed statistical analysis, and drafted manuscript. ZZ contributed to statistical analysis, data interpretation and manuscript writing. ZX contributed to data interpretation and manuscript writing. FN contributed to statistical analysis and data interpretation. MB, PA, PAB, and AA planned the study subject's recruitment and exposure assessment. JS supervised statistical strategies and contributed to data interpretation. MH and LD performed the laboratory analyses. VP managed the research database and performed statistical analysis. All authors read and approved the final manuscript.
